# Patellar Tendon Reconstruction for Chronic Patellar Tendon Rupture Using Hamstring Tendons: The Reverse Double Figure-of-Eight Technique

**DOI:** 10.1016/j.eats.2025.103851

**Published:** 2025-08-30

**Authors:** Dany Mouarbes, Ali Alayane, Victor Sonnery-Cottet, Regis Pailhe, Etienne Cavaignac

**Affiliations:** a Clinique Universitaire du Sport, Centre Hospitalier Universitaire de Toulouse (CHU), Toulouse France; b Centre Hospitalier de Perpignan (CHP), Perpignan, France; c Cabinet Arthropole, Bayonne, France

## Abstract

Chronic patellar tendon rupture has devastating functional consequences attributable to the loss of the extensor mechanism. Repairing a neglected patellar tendon rupture is challenging and often nearly impossible because of proximal patellar retraction and the poor quality of the remaining tendon. In this Technical Note, we describe a surgical technique for chronic patellar tendon reconstruction using semitendinosus and gracilis tendon autografts. Both tendons are left attached to the tibia and passed in a reverse figure-of-eight configuration to reconstruct the patellar tendon. The remaining viable patellar tendon tissue is reinserted into the tibia using a 5.5-mm anchor to restore the patellar height and enhance graft healing.

Patellar tendon (PT) ruptures are most frequently seen in active individuals younger than 40 years of age.^1^ Tendon rupture is typically a result of chronic degeneration caused by repetitive microtrauma to the knee, often triggered by an acute injury, and it usually occurs unilaterally.^2^^,^^3^ This rupture disrupts the continuity of the extensor mechanism, leading to poor functional outcomes.^4^ Neglected PT rupture is defined as a tendon rupture that has remained untreated for 6 weeks or longer. These chronic PT ruptures are relatively uncommon, leading to limited research on the topic and an unclear understanding of their true incidence.^5^ Surgical repair of a chronic PT rupture is typically a challenging procedure with a high failure rate as a result of factors such as poor quality of the remaining tendon tissue, associated adhesions, and proximal patellar migration.^6^ Many surgical techniques that aim to restore patellar height and achieve full knee joint extension^7^, ^8^, ^9^ have been described for the reconstruction of chronic PT rupture. PT reconstruction involves the use of either bone−PT−bone or hamstring autografts^6^^,^^8^ as well as allografts.^9^^,^^10^ In this Technical Note, we describe a surgical technique for reconstruction of chronic PT rupture using ipsilateral hamstring tendon autografts (Video 1). Both tendons are kept attached to their tibial insertion to provide biological PT reconstruction and enhance healing. The patellar height is restored by reinserting the PT remnants to the tibia using a 5.5-mm suture anchor (Fixit; SBM France). Surgical pearls and pitfalls of the procedure are described in Table 1. Advantages and disadvantages are presented in Table 2.

**Table 1:** Pearls and Pitfalls of the Procedure

Pearls	Pitfalls
All fibrotic tissues should be removed from the patellar tendon to allow optimal biological healing of the graft and the patellar remnants.	Avoiding patellar fracture or cartilage damage by drilling the tunnel through the middle part of the patella
The separation of hamstring tendons at their tibial insertion after harvesting provides extra length for the reconstruction.	To avoid reconstruction failure, patellar height should be restored with the knee in full extension by reinserting the patellar tendon remnants at the lateral border of the tibial tuberosity before final graft fixation in the tibial tunnel using an interference screw.
Both tendons should be passed in a figure-of-eight configuration through the patellar defect and under the medial and lateral patellar retinaculum before passing through the patellar tunnel to achieve an anatomical and biological patellar tendon reconstruction.

**Table 2:** Advantages and Disadvantages of the Procedure

Advantages	Disadvantages
The hamstring grafts are passed in a figure-of-eight configuration within the patellar tendon allowing for a robust and anatomically accurate reconstruction of the patellar tendon.	Risk of failure to restore patellar height, leading to poor functional outcomes.
The hamstring tendons are kept attached to the tibia, allowing biological graft healing.	Risk of hamstring tendon weakness.
Patellar tendon reconstruction using hamstring autografts, rather than allografts, reduces the risk of complications typically associated with allografts such as infection and graft failure.	Not feasible in cases with poor-quality tendon remnants.
Unlike the bone−patellar tendon−bone graft technique, the patellar bone stock is preserved without the need for screw fixation, resulting in a lower risk of patellar fracture.	Requires a moderate learning curve.

## Surgical Technique

### Patient Setup

**Fig 1:**
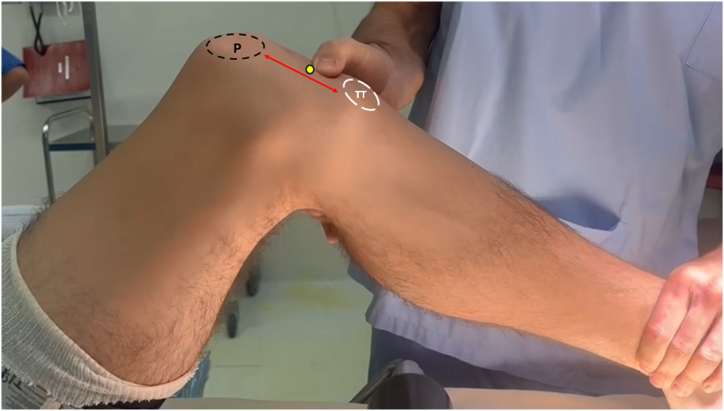
Intraoperative photograph of the left knee showing the defect within the patellar tendon (yellow circle) associated with proximal migration of the patella. Red arrow: patellar height. (P, patella, TT, tibial tuberosity.)

Under general anesthesia, the patient is placed in a supine position with a tourniquet applied to the operative thigh. The foot is positioned on padded supports, allowing for a full assessment of knee range of motion and stability at 90° flexion. Preoperative evaluation of patellar height is performed to assess the extensor mechanism deficiency. A palpable defect in the PT confirms its chronic rupture (Fig 1).

### Surgical Approach and Fibrotic Tissue Excision

**Fig 2:**
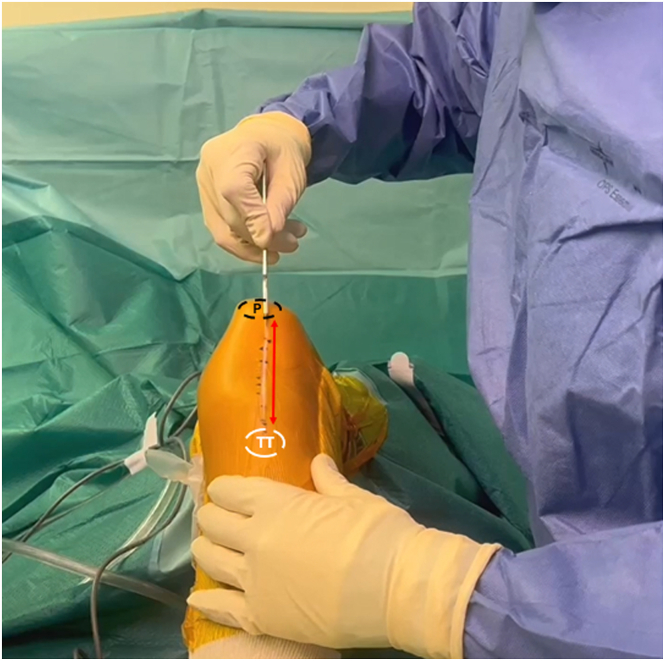
Intraoperative photograph of the left knee showing the anterior midline skin incision (red arrow), which begins from the tibial tuberosity to the lower border of the patella. (P, patella; TT, tibial tuberosity.)

**Fig 3:**
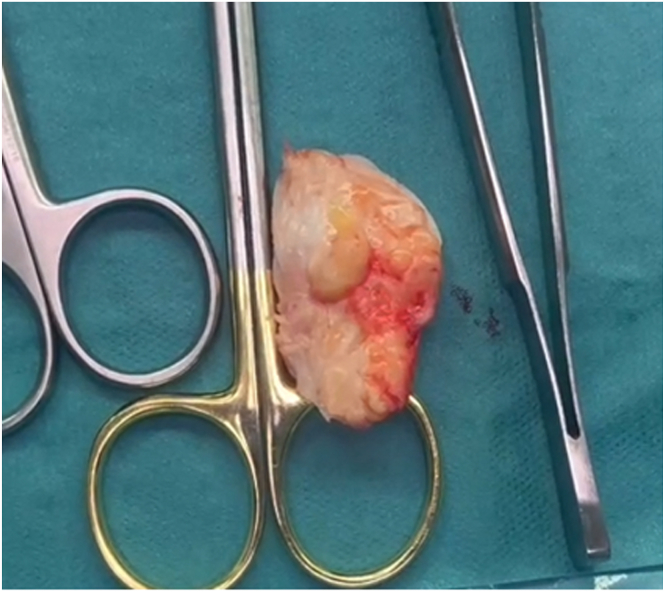
Intraoperative photograph showing the excised fibrotic tissues from the patellar tendon body.

**Fig 4:**
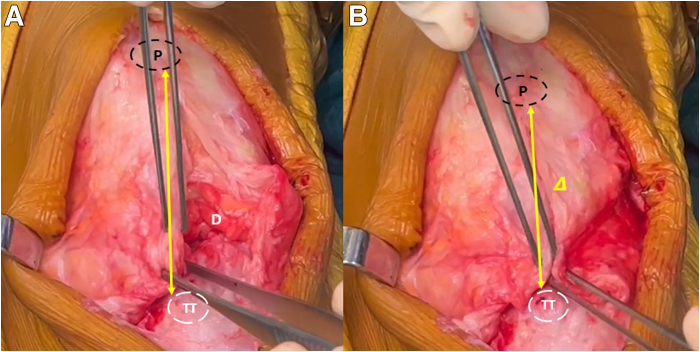
Intraoperative photograph of the left knee showing the patellar tendon remnants and the defect after excision of fibrotic tissue with proximal migration of the patella (A). Reinsertion of the patellar remnants allows restoration of the patellar height (B). Yellow arrow: Patellar height; delta shape: difference in the patellar height before and after reinsertion of the patellar remnants. (D, patellar defect, P, patella; TT, tibial tuberosity.)

A midline anterior left knee joint skin incision is made from the upper border of the patella to the tibial tuberosity (Fig 2). Two subcutaneous flaps are created to expose the patella and the PT remnants. Fibrotic tissues and adhesions (Fig 3) are excised from the PT and the viable PT remnants are debrided (Fig 4A) to be reinserted at the tibial tuberosity during the final stage of the procedure (Fig 4B).

### Hamstring Tendon Harvesting

**Fig 5:**
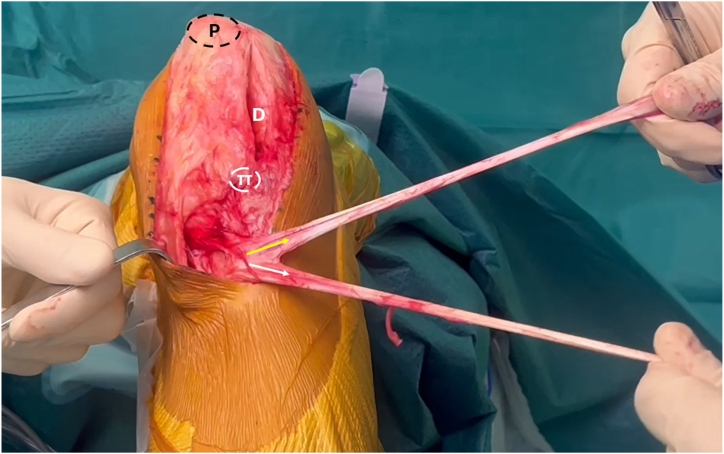
Intraoperative photograph of the left knee, showing the harvested semitendinosus (white arrow) and gracilis (yellow arrow) tendons. Tendons extremities are secured with No. 2 nonabsorbable suture and remain attached to the tibial tuberosity. (D, patellar defect, P, patella; TT, tibial tuberosity.)

The semitendinosus and the gracilis tendon are harvested in the standard manner, and they are kept attached to their tibial insertion (Fig 5). The 2 ends of both tendons are secured with No. 2 nonabsorbable suture.

### Patellar and Tibial Tunnel Creation

A guidewire is inserted through the patella at its middle border, from lateral to medial, and overdrilled with a 5-mm drill bit to create a transverse patellar tunnel. A loop sutured is then passed through the tunnel.

A transverse tibial tunnel is created by inserting a guidewire 1 cm posterior to the tibial tuberosity and overdrilling it from lateral to medial using a 5-mm diameter drill bit. A loop suture is the passed and secured within the tunnel.

### Hamstring Tendon Graft Passage

**Fig 6:**
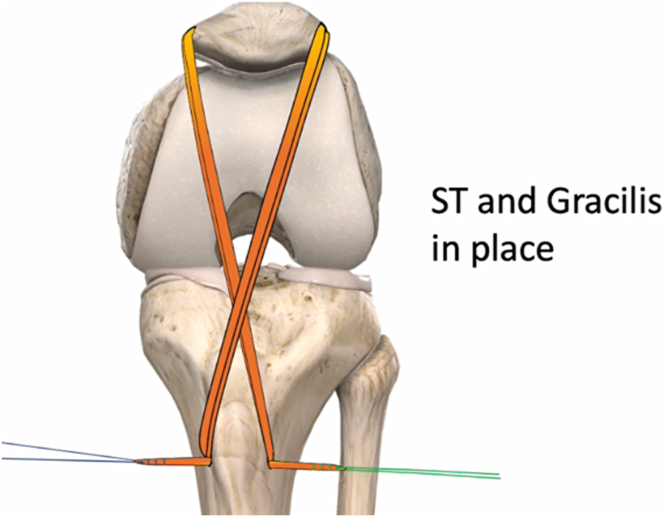
Schematic illustration showing the passage of the semitendinosus and gracilis grafts in a reverse figure-of-eight configuration through the patellar defect and beneath medial and lateral patellar retinaculum before final fixation within the tibial tunnel.

The hamstring tendons are preserved at their tibial insertion and passed in a reverse figure-of-eight configuration through the patellar and tibial tunnels (Fig 6). The gracilis tendon is first passed under the lateral retinaculum and then through the patellar tunnel from lateral to medial. It is subsequently passed beneath the medial reticulum, crossing over the PT, before being directed through the tibial tunnel from lateral to medial.

Similarly, the semitendinosus tendon is passed through the tibial tunnel from medial to lateral direction. However, it follows the reverse course of the gracilis tendon, passing through the patellar tunnel from medial to lateral, crossing the PT and existing through the tibial tunnel from medial to lateral (Video 1).

### PT Reinsertion and Hamstring Graft Fixation

**Fig 7:**
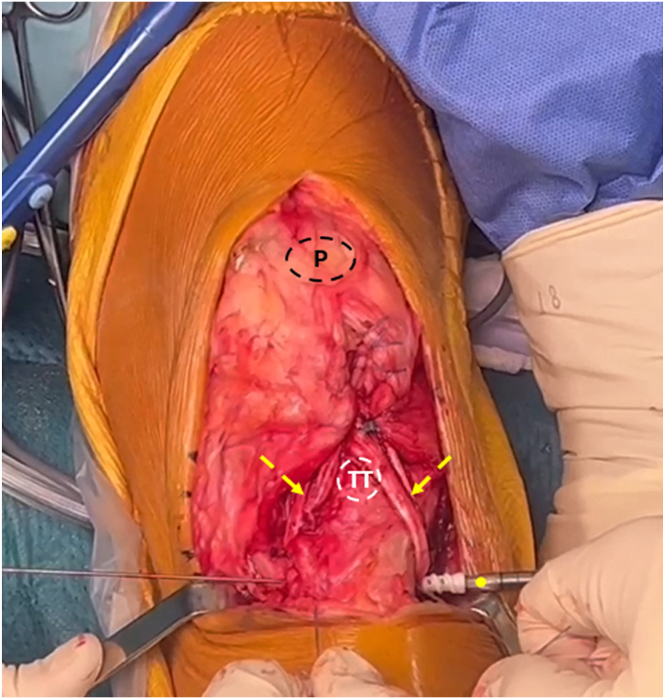
Intraoperative photograph of the left knee showing the tibial fixation of the hamstring tendons using an interference screw (yellow circle) after restoration of patellar height and the reinsertion of the patellar tendon remnants onto the tibial tuberosity. Yellow arrows: semitendinosus and gracilis grafts in a figure-of-eight configuration. (P, patella; TT, tibial tuberosity.)

A 5.5-mm suture anchor (Fixit) is inserted into the lateral border of the tibial tuberosity, and the PT remnants are prepared using the Krakow technique. The viable remnants are then reinserted into the tibial tuberosity with the knee in full extension to restore the patellar height, which can be confirmed with intraoperative radiographs. The grafts are subsequently tensioned and secured within the tibial tunnel using a 6-mm interference screw (Fig 7).

### Postoperative Protocol

The postoperative protocol involves knee immobilization for 6 weeks and immediate weight-bearing with crutches. Physiotherapy includes progressive range-of-motion exercises to restore full knee extension and improve the quadriceps muscle contraction (Video 1). Return to sports is allowed after 4 months, starting with nonpivoting activities and progressing to pivoting sports for 6 months postoperatively.

## Discussion

Extensor mechanism injury can lead to considerable functional impairment, mainly in active individuals.^11^ Direct repair of chronic PT rupture is uncommon because of quadriceps retraction and a lack of viable tissue.^12^

This Technical Note describes a surgical technique for reconstruction of chronic PT rupture using hamstring tendon autografts. Hamstring autografts are used instead of contralateral bone−PT−bone autograft or allograft aiming to minimize the associated complications related to these techniques such as anterior knee pain, patellar fracture, and nonunion of bone plugs. In addition, the hamstring tendons are left attached to their tibial insertion to preserve tendon vascularity, thereby enhancing graft healing.^13^ The grafts are passed in a reverse figure-of-eight configuration through patellar and the tibial tuberosity tunnels, providing an optimal biological and anatomical patellar tendon reconstruction.

The key step of this technique is the restoration of the patellar height, achieved by reinserting the viable PT remnants onto the tibial tuberosity with a 5.5-mm suture anchor before final graft tensioning and fixation within the tibial tunnel in full knee extension. This ensures proper extensor mechanism restoration and reduces the risk of reconstruction failure. However, the absence of viable PT tissue makes this technique not feasible. From our perspective, this technique provides a reliable approach to reconstruct the extensor mechanism in young patients with chronic PT insufficiency, using a unilateral hamstring autograft and achieving favorable functional outcomes.

## Disclosures

The authors declare the following financial interests/personal relationships which may be considered as potential competing interests: E.C. reports consulting or advisory with Arthrex. All other authors (D.M., A.A., V.S-C., R.P.) declare that they have no known competing financial interests or personal relationships that could have appeared to influence the work reported in this paper.

## Supplementary Data

Video 1 This instructional video demonstrates the surgical technique for patellar tendon reconstruction in a case of chronic rupture of the left patellar tendon. The patient is placed in a supine position and a tourniquet is applied to the left thigh. Preoperative planning involves both clinical examination and radiographic evaluation of the patellar height and the degree of the tendon defect. A midline anterior knee incision is performed, and extensive fibrotic tissue excision is performed to identify and preserve viable PT remnants. The semitendinosus and gracilis tendons are harvested while preserving their tibial insertions to promote biological integration and graft viability. The harvested tendons are passed in a reverse double figure-of-eight configuration through previously created transverse tunnels in the patella and the tibial tuberosity. The patellar tendon remnants are prepared and reinserted at the lateral border of the tibial tuberosity using a 5.5-mm suture anchor (Fixit; SBM France), with the knee maintained in full extension to ensure restoration of proper patellar height. The grafts are then tensioned and fixed within the tibial tunnel using a 6-mm interference screw. The goal of this procedure is to provide a biological reconstruction of the patellar tendon and restore appropriate patellar height, thereby reestablishing a functional extensor mechanism.
